# Synergism between TLR4 and *B. infantis* in the development of the premature intestine

**DOI:** 10.1038/s41390-024-03676-5

**Published:** 2025-02-12

**Authors:** Wuyang Huang, Karim Djebali, Ky Young Cho, Kimberly Gardner, Alessio Fasano, Di Meng, W. Allan Walker

**Affiliations:** 1 Institute of Agro‑Product Processing, Jiangsu Academy of Agricultural Sciences, Nanjing, People’s Republic of China; 2 Mucosal Immunology and Biology Research Center, Massachusetts General Hospital for Children, Harvard Medical School, 16th Street Building (114‑3503), Charlestown, MA 02129 USA; 3 Department of Microbial Diseases, Eastman Dental Institute, University College London, Royal Free Campus, Rowland Hill Street, London, NW3 2PF UK; 4 Department of Pediatrics, Hallym University Kangnam Sacred Heart Hospital, Seoul, South Korea; 5 Howard University, 2400 6th ST NW, Washington, DC 20059 USA

## Abstract

**Background:**

Intestinal microbiota has a role in early life maturation including maturation of intestinal immune function. However, the interaction of the TLR4 with colonizing bacteria in intestinal development is incompletely understood.

**Methods:**

An established human immature small intestinal cell line, human fetal intestinal organoids, and wild-type (WT) and TLR4 gene knockout (TLR4 ^−/−^) neonatal mice were used to test the synergism between the innate immune receptor TLR4 and postbiotics from *Bifidobacteria longum* subsp. *infantis* (*B. infantis*) in development of the premature intestine.

**Results:**

TLR4-mediated postbiotics induced immature enterocyte proliferation and filamentous actin (F-actin) maturation both at the mRNA and protein levels. Proliferation of mRNA levels increased in wild-type mice but not in TLR4 ^−/−^ mice fed by postbiotics, both in the ileum and colon. Postbiotics can also change tight junction distribution in WT neonatal colon but not in TLR4 ^−/−^ mice.

**Conclusions:**

Our data suggest a novel regulation of intestinal development by a synergistic role of the innate immune receptor TLR4 and early life colonizing bacteria, such as *B. infantis*. This study should provide new insights into the mechanisms of intestinal maturation as well as opportunities to target novel approaches to NEC prevention and treatment.

**Impact:**

- The innate immune system and postbiotics affect immature intestinal development.
- The innate immune receptor TLR4 prevention of NEC.
- Mechanism of prevention of NEC.
- This is the first time this has been demonstrated in human fetal intestine.
- In vitro process for future clinical studies for prevention of NEC.

## Introduction

Previous studies have shown that premature infants have incomplete development of the gastrointestinal tract that may account for conditions such as necrotizing enterocolitis (NEC) which increases morbidity and mortality.^1–4^ We and others have shown experimentally and clinically that the combination of preterm breast milk and colonizing bacteria protects the immature intestine from excessive inflammation and stimulates enterocyte development, potentially overcoming two key risks associated with these immaturities.^5–11^ The most common colonizing bacteria in term infants, especially in breastfed infants, is *Bifidobacterium longum*, subspecies *infantis (B. infantis)*.^2^ However, since this is not necessarily the case with premature infants and the breastmilk they obtain, added supplement may be necessary. We have previously shown that TLR4 acts as an innate immune receptor for both colonizing bacteria and their postbiotics, the bioactive compounds the probiotic bacteria produce when they consume prebiotics, leading to either pro- or anti-inflammatory signals. However, little is known about its role as a developmental receptor, a function that has been previously demonstrated in the species *Drosophila* during maturation.^12–15^ Accordingly, this study seeks to define the possible mechanism(s) by which the TLR4 receptor contributes to gastrointestinal maturation in vitro using a human fetal cell line, H4 cells,^16^ authenticated by human fetal gut organoids, as well as in vivo in mouse pups. To do so, we have examined representative developmental genes and proteins in both wild-type and TLR4 knockout models. Specifically, both in premature human and murine models, we have used the *Ki67* gene as a biomarker of cell proliferation^17^ and the zonula occludens-1 (ZO-1), also known as tight junction protein 1 (TjP1), as a biomarker of intercellular tight junction formation and function, regulation of epithelial cell proliferation, gene expression, differentiation, and morphogenesis.^18,19^ In the in vivo mouse model, we also used several genes involved in cell cytoskeleton formation and dynamic as additional biomarkers of cell proliferation and maturation. Finally, using the biomarkers mentioned above, we have investigated whether postbiotics from *B. infantis* in breast milk or as a probiotic stimulate epithelial cell proliferation and, if so, whether this proliferative effect is TLR4-dependent.

## Methods

### Chemicals used

Dulbecco’s modified Eagles medium (DMEM), Opti-MEM I medium, MEM nonessential amino acid (NEAA, 100×), glutamine (100×), antibiotic-antimycotic (100×), sodium pyruvate (100 mM), HEPES [4-(2-hydroxyethyl)-1-piperazineethanesulfonic acid] buffer (1 M), Trizol reagent, and the BCA (bicinchoninic acid) Protein Assay Kit were obtained from Thermo Fisher Scientific (Waltham, MA). Rnase-free Dnase kits and RNeasy Mini kits were purchased from Qiagen (Frederick, MD). Fetal bovine serum (FBS) was obtained from Atlanta Biologicals (Flowery, GA). Humulin R (insulin human recombinant) (100 units/mL) was obtained from Eli Lilly and Company (Indianapolis, IN). Tissue culture plastics were obtained from Fisher Scientific (Pittsburgh, PA). All-in-one cDNA Synthesis SuperMix was purchased from Bimake.com (Houston, TX) and 2x High ROX Apex qPCR GREEN Master Mix was purchased from Genesee Scientific (EI Cajon, CA). TLR4 Stealth RNAi™, siRNA Negative Controls and lipofectamine RNAiMax were obtained from Thermo Fisher Scientific (Waltham, MA). Antibodies for immunostaining were purchased from the companies as following: Ki67 and 4’6-diamidino-2-phenylindole (Dapi) from Cell Signaling Technology (Danvers, MA); Fluorescent-isothiocyanate (FITC) labeled Phalloidin from Sigma (St. Louis, MO); Cy^TM^3 conjugated AffiniPure donkey-anti-rabbit Ig G (H + L) from Jackson ImmunoResearch Lab (West Grove, PA); goat anti-mouse IgG-FITC from Santa Cruz Biotechnology (Dallas, TX); Avidin/Biotin blocking kit from Vector Laboratories (Burlingame, CA); Fluoromount G™ Mounting Medium from ThermoFisher Scientific-Invitrogen (Carlsbad, CA); and TLR4 inhibitor ethyl (6 R)-6-[N-(2-Chloro-4-fluorophenyl) sulfamoyl] cyclohex- 1-ene-1-carboxylate (TAK-242) from Sigma Aldrich. All the chemicals and reagents were analytical grade.

### Bacterial cultures and postbiotic preparations

*Bifidobacterium longum* subspecies *infantis (B. infantis)* (ATCC No. 15697) was obtained from American Type Culture Collection (ATCC, Manassas, VA) and were cultured anaerobically in a media modified from the combination of Mann-Rogosa-Sharpe (MRS) broth (DIFCO; BD Bio-science, Franklin Lakes, NJ) and H4 cell culture media as described in our previous publication.^10^ Postbiotics were prepared by centrifugation of *B. infantis* cultures at a stationary growth phase (OD600 > 1.0) at 3700 rpm (equal 2936 g) (Sorvall legend RT+ centrifuge, ThermoFisher Scientific, MA) for 10 min and then by 0.22-μm filtration to eliminate residual bacteria, followed by storage at −80 °C for later use.

### Cell cultures and treatments

H4 cells, a human fetal non-transformed primary intestinal epithelial cell line, were characterized by our laboratory^16^ with approval from the committee for human studies at Massachusetts General Hospital (IRB 2018-P002987) and used as an in vitro model of the immature intestine. The cells were cultured in DMEM supplemented with 10% FBS (heat-inactivated), 1% NEAA, 2 mM *L*-glutamine, 1% antibiotic-antimycotic solution, 10 mM HEPES buffer, 1 mM sodium pyruvate, and 0.2 units/mL human recombinant insulin. H4 cells were transfected with TLR4 siRNA or control siRNA to make TLR4 gene knock down (TLR4KD) and control wild-type (WT) H4 cells as described in our previous publication.^12^ Briefly, TLR4 stealth RNAi 12.5 pmol and 2 μL of lipofectamine siRNAMax were added to 200 μL of Opti-MEM only media (no antibiotics, no serum), then added to a 12-well plate for 20 min at room temperature (RT), followed by adding 300 μl of H4 cells in antibiotic-free cell culture media to reach 1.5–2 × 10^5^ cells/25 nM siRNA/well. The second day, an additional 0.5 mL antibiotic-free H4 culture media was added to each well to bring the final concentration of siRNA to 12.5 nM. The cells were used for all experiments at 80–90% confluence. H4 (WT and TLR4KD) cells were seeded on 6-well/12-well plates and incubated with or without 10% of postbiotics (e.g.100 μl of postbiotics in 900ul of cell culture media in a 12-well plate) from a storage pod of *B. infantis* for 24 h.

### Animals and treatments

C57BL/6 WT and C57BL/6 background TLR4^−/−^ mice obtained from Jackson Laboratory were housed in a specific pathogen-free facility. Animal procedures had been previously approved by the Massachusetts General Hospital Subcommittee on Research Animal Care and Use committee (2018N000070). Animals were given water and standard laboratory chow *ad libitum*. Littermate newborn pups were randomly divided into control (culture media) and postbiotics (*B. infantis* conditional media) groups. Each pup at postnatal day 4 (P4) received 5 μL of postbiotics or culture media via gavage feeding once on the first day followed by 7 μL twice per day for 3 days and then 7 μL once on the fifth day. At the end of the experiments, all pups in each group were euthanized and the intestinal ileum and colon collected and cut into 3-mm pieces for organ culture. In addition, some intestinal tissues were fixed in 4% paraformaldehyde for histological assessment and some were stored at −80 °C for total RNA isolation. Organ culture media was prepared as described previously. ^12,13^ Briefly, Opti-MEM I medium supplemented with 10% FBS, 2 mM *L*-glutamine, 10 mM HEPES buffer, 0.2 U/mL insulin, and 20 ng/mL recombinant mouse EGF, 5 μg/mL transferrin, 0.06 μM sodium selenite, 200 nM hydrocortisone, and an 1% antibiotic-antimycotic cocktail (100 unit/mL penicillin, 100 μg/mL streptomycin, and 0.25 μg/mL fungizone antimycotic) were used.

### Total RNA isolation

A Trizol/RNeasy hybrid protocol was used for total RNA isolation. H4 cells were dissolved in 1 mL of Trizol in a 2 mL Eppendorf tube and incubated at room temperature for 5 min, then 0.2 mL of chloroform was added. The tubes were then shaken vigorously for 20 s and kept at room temperature (RT) for 5 min, followed by a centrifugation at 14,000x rpm for 30 min at 4 °C. The upper layer of the supernatant was transferred to a new tube and a volume of 70% alcohol was added. The procedure of Qiagen RNeasy Mini Kit for total RNA isolation was followed by a DNase I step to remove any remaining DNA to get total RNA. The total RNA was dissolved in RNase free water and kept in −80 °C for later use. Experiments were performed in triplicate. Mouse ileum and colon were homogenized in Trizol then the same procedure as described above was followed to obtain the total RNA. The quantity of the extracted RNA was measured by Nano Drop ND-1000 spectrophotometer (Thermo Scientific, Wilmington, DE/Rockford, IL). Only RNA samples with A_260_/A_280_ and A_260_/A_230_ ratios of 1.8–2.0 were used for further analysis.

### Real-time quantitative reverse transcription PCR (qRT-PCR) analysis

RNA was reverse transcribed with an All-in-one cDNA Synthesis SuperMix kit. The cDNA was amplified using 2x High ROX Apex qPCR GREEN Master Mix. The genes and their primer sequence in qRT-PCR are shown in Table 1. qRT-PCR experiments were carried out in a 96-well plate with an Applied Biosystems StepOnePlus Real-Time PCR system (Thermo Fisher Scientific Inc., San Diego, CA). GAPDH primers were amplified in all samples to represent a housekeeper gene. Triplicate cDNA samples were amplified with the primers, mean threshold cycle (Ct) values of each transcript were normalized by subtracting the mean Ct value for the GAPDH transcript of that sample. Relative gene expression data was analyzed using the 2^-ΔΔCt^ method. ∆Ct = Ct (gene of interest) – Ct (housekeeping gene), ∆∆Ct = ∆Ct (treated sample) – ∆Ct (untreated control sample). All data were expressed as fold increase over the corresponding control.

**Table 1:** Primers for gene analysis.

Gene Name	Species	Primer sequences (5′-3′)	
Ki67	Human	forward	GGGCCAATCCTGTCGCTTAAT
		reverse	GTTATGCGCTTGCGAACCT
TERT (Telomerase reverse Transcriptase)	Human	forward	CCGATTGTGAACATGGACTACG
		reverse	CACGCTGAACAGTGCCTTC
GAPDH (glyceraldehyde 3-phosphate dehydrogenase)	Human	forward	ATGGGGAAGGTGAAGGTCG
		reverse	GGGGTCATTGATGGCAACAATA
Tubulin-β3	Mouse	forward	GCGCCTTTGGACACCTATTCA
		reverse	GCCCTCCGTATAGTGCCCT
β-Actin	Mouse	forward	AGTGTGACGTTGACATCCGTA
		reverse	GCCAGAGTAATCTCCTTCT
Krt18 (keratin 18)	Mouse	forward	CAGCCAGCGTCTATGCAGG
		reverse	CCTTCTCGGTCTGGATTCCAC
Krt20 (keratin 20)	Mouse	forward	CAACGGATCGGACCTGTTTG
		reverse	AGCGCACTTTTTCTAGGTAGTTT
Ki67	Mouse	forward	GGGCCAATCCTGTCGCTTAAT
		reverse	GTTATGCGCTTGCGAACCT
TjP1 (tight junction protein 1)	Mouse	forward	GAGCGGGCTACCTTACTGAAC
		reverse	GTCATCTCTTTCCGAGGCATTAG
GAPDH (glyceraldehyde 3-phosphate dehydrogenase)	Mouse	forward	ATGACCTTGCCCACAGCCT
		reverse	CCTGCACCACCAACTGCTTA

### Human organoid cultures

Use of human samples was previously approved by the Mass General Brigham IRB (protocol number: 2018P 002606) and Partners Human Research Committee (Protocol No. 1999P 003833 for the derivation of immature enterospheres (FEnS). Human immature intestinal organoids were derived as described in a previous publication.^20^ In this study, the FEnS were from gestational aged 15-week and 21-week-old fetuses with similar results (data from a 21-week-old fetus are not shown). TLR4 Stealth siRNA and its control siRNA were transfected into the FEnS cells immediately before the cells were seeded on the upper side of a trans well membrane. Seventy-two hours after siRNA transfection, total RNA was isolated and mRNA determined by qRT-PCR. The monolayer derived from the non-transfected FEns (F15) was pretreated with the TLR4 inhibitor TAK242 in culture media at the beginning of the cell seeding. Following that, the cells were treated with 10% postbiotics for 20 h when they reached more than 90% confluence. Paracellular permeability was measured by FITC-Dextran (4-kilodalton) passage to the basolateral side after 2 h of incubation by determining the OD value at 450 nm wavelength.

### Statistical analysis

All data are presented as the mean ± standard error of the mean (SEM). Quantitative analyses of the immunofluorescent intensity were measured on the ROIs in ImageJ software (National Institutes of Health, Bethesda, MD). Figures were obtained by using GraphPad Prism Version 7 (GraphPad Software, Inc., CA). One-way analysis of variance (ANOVA) with a turkey’s post hoc test or two-way ANOVA was used to compare the mean of multiple groups. A repeated test was used wherever applicable. *p* < 0.05 were considered a significant differences (*p < 0.05, **p < 0.01, ***p < 0.001).

## Results

### Postbiotics and the TLR4 receptor increase hTERT and Ki67 mRNA expression in H4 cells

**Fig. 1:**
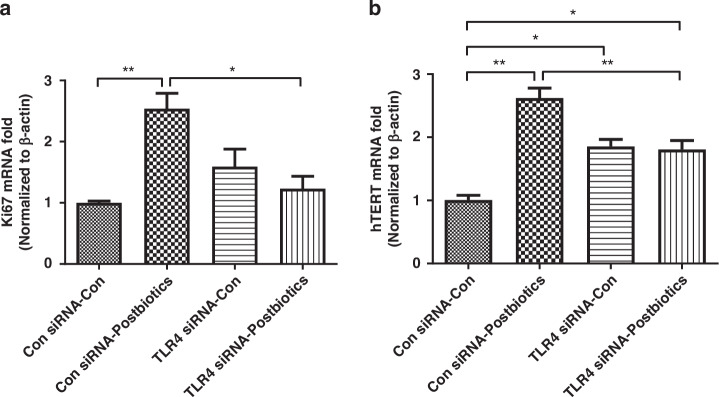
TLR4 mediates *B. infantis* postbiotic-induced Ki67 and hTert mRNA in H4 cells. H4 cells transfected with TLR4 siRNA or control siRNA were treated with or without postbiotics from *B. infantis*. Total RNA was isolated for the proliferation related genes Ki67 (**a**) and Telomerase reverse transcriptase (hTERT) (**b**) mRNA was determined by qRT-PCR. Data are mean ± SEM, *n* = 3, representative of three independent experiments. *P < 0.05, **P < 0.01.

WT H4 cells exposed to *B. infantis* postbiotics showed a significant increase in Ki67 (Fig. 1a) and hTERT (Fig. 1b) mRNA expression. These genetic upregulations were prevented in TLR4KD H4 cells (Fig. 1). In TLR4KD H4 cells, the baseline expression of hTERT mRNA was significantly higher compared to baseline expression in control H4 cells (Fig. 1b).

### Postbiotics increase Ki67 and F-actin protein expression in H4 cells

**Fig. 2:**
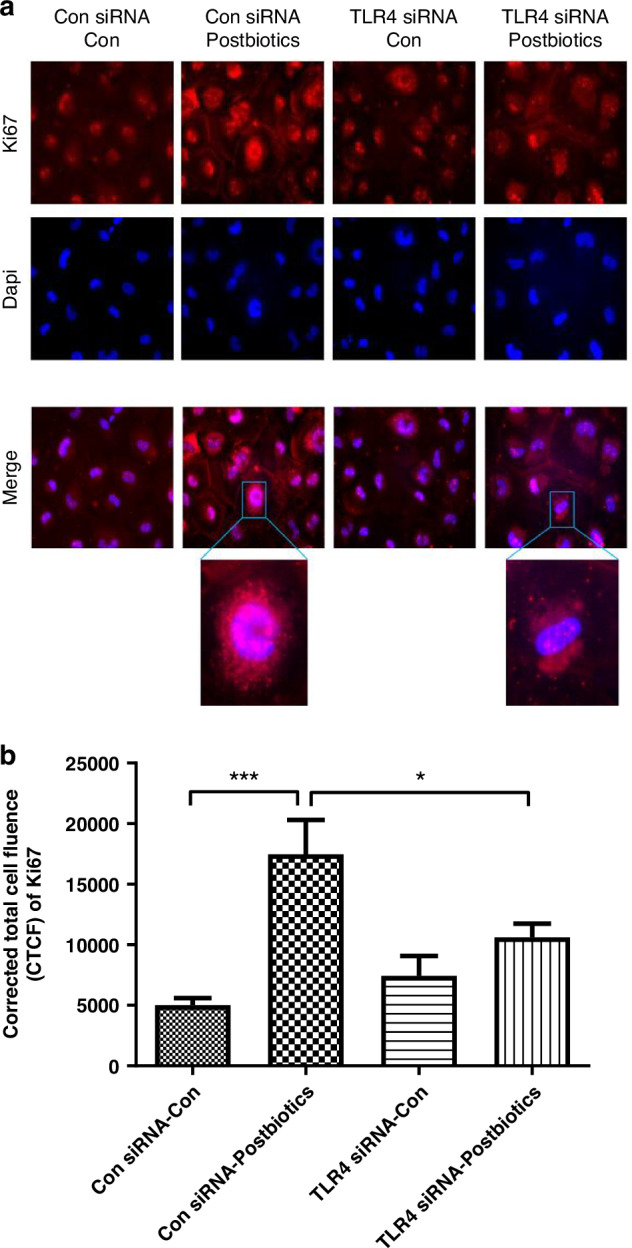
TLR4 is required for postbiotics from *B. infantis-* induced Ki67 protein induction in H4 cells. H4 cells transfected with TLR4 siRNA or control siRNA were seeded onto chamber slides and treated with or without postbiotics from *B. infantis*. Ki67 was detected by immunofluorescent (IF) staining (red), the nuclei were counterstained with Dapi (blue) (**a**). The images are representative of 3 separate experiments with similar results. The analysis applied by corrected total cell fluorescence (CTCF) with image J (**b**). One-way ANOVA and post hoc tests were used for statistic-analysis, *P < 0.05, ***P < 0.001. Amplified x 400; scale bar = 100 pixels; *n=* 11 cells/treatment.

**Fig. 3:**
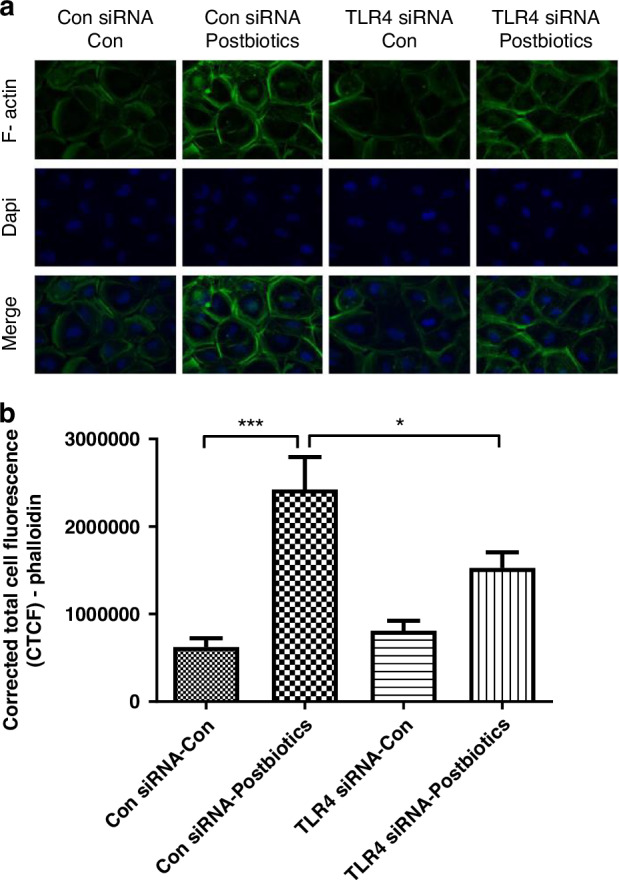
TLR4 regulates the effect of postbiotics from *B. infantis* on F-actin protein expression. H4 cells transfected with TLR4 siRNA or control siRNA were seeded onto chamber slides and treated with or without postbiotics from *B. infantis*. F-actin was detected by IF staining (green), the nuclei were counterstained with Dapi (blue) (**a**). The images are representative of 3 independent experiments. The analysis applied by corrected total cell fluorescence (CTCF) with image J (**b**). One-way ANOVA and post hoc tests were used for statistic-analysis, *P < 0.05, ***P < 0.001. Amplified x 400; scale bar = 100 pixels; *n=* 10–17 cells/treatme*n*t.

We further explored Ki67 expression at the protein level by immunofluorescent staining in control and TLR4KD H4 cells at baseline and after exposure to postbiotics. Postbiotic exposure significantly increased Ki67 protein expression in WT H4 cells but not in TLR4KD H4 cells (Fig. 2). Ki67 protein was present in the nucleus of proliferative cells, in phase G1, S, G2, and M, but not in the G0 phase. After postbiotic stimulation, Ki67 was localized mostly in the nuclear area (Fig. 2) in control H4 cells, but not in TLR4KD H4 cells. We further investigated cytoskeleton modifications by examining F-actin in H4 cells. We exposed WT and TLR4KD H4 cells to postbiotics and detected F-actin protein by immunofluorescent staining. The result showed that postbiotics significantly increased F-actin protein expression in control but not in TLR4KD H4 cells (Fig. 3).

### TLR4 plays a role in postbiotic-mediated human fetal intestinal organoid-derived monolayer proliferation, tight junction gene expression and permeability

**Fig. 4:**
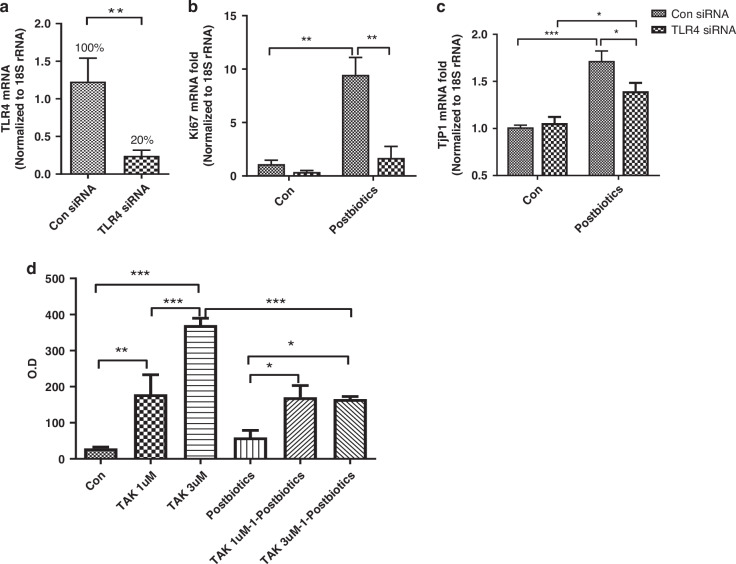
The interaction of TLR4 and postbiotics on human fetal intestinal organoid growth (gestational age 15 week). (**a**) TLR4 gene knockdown efficacy in human fetal-derived enterospheres (FEnS). The results were normalized to 18SrRNA. The data represent the combination of two experiments, *n* = 8 for each experiment. TLR4 gene knockdown effect on postbiotics-induced Ki67 (**b**) and Tjp1 mRNA (**c**) induction in the FEnS-derived monolayer. The fold change of the gene was normalized to housekeeper gene 18S rRNA. The data are representative of three independent experiments, results are expressed as means ± SEM (*n* = 3). (**d**) Different concentrations (1 and 3 μM) of TLR4 inhibitor TAK242 increased the O.D level of the trans-epithelial collection of 4 kDa fluorescein isothiocyanate (FITC)-dextran in a human fetal intestinal organoid-derived monolayer inhibited by degrees of TLR4. The comparison analysis was performed with one-way ANOVA and Tukey multiple comparison tests. *p < 0.05, **p < 0.01, ***p < 0.001.

To authenticate the data obtained in the H4 cell line, we performed similar representative developmental experiments using fetal intestinal organoids under baseline conditions and after TLR4 silencing or inhibition. TLR4 expression was decreased by 80% in TLR4 siRNA 15-week-old organoids (Fig. 4a). TLR4 silencing did not change the baseline expression of Ki67, while it was highly increased by probiotic treatment in TLR4 expressing organoids but not in TLR4-silenced organoids (Fig. 4b). TjP1 expression was also TLR4-independent at baseline but stimulated by probiotics both in TLR-expressing organoids and in TLR-deficient organoids, even if it was significantly lower compared to TLR-expressing organoids (Fig. 4c). Similar data were obtained using 21-week-old human fetal organoids (data not shown).

In addition, a TLR4 inhibitor TAK-242 significantly increased the O.D level of the trans-epithelial collection of 4 kDa fluorescein isothiocyanate (FITC)-dextran in a human fetal intestinal organoid-derived monolayer (Fig. 4d). Probiotics significantly decreased the O.D level induced by a high concentration of TAK-242 but not for a low concentration of TAK-242.

### TLR4 regulates postbiotic-induced Ki67 mRNA and TjP1 mRNA in mouse pups

**Fig. 5:**
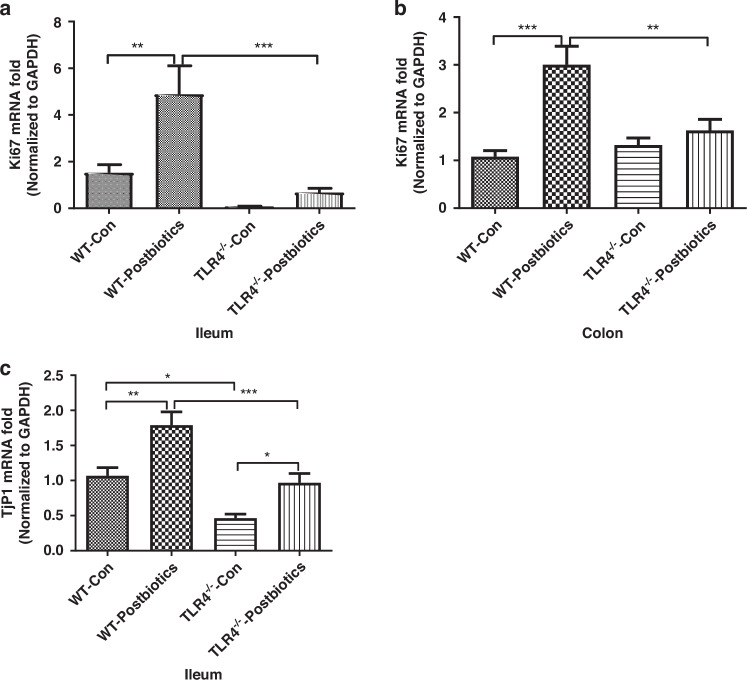
TLR4 regulates *B. infantis* postbiotic-induced Ki67 and TjP1 mRNA induction in mouse pups. **a** Different effects of postbiotics on Ki67 mRNA expression in C57-pup and TLR4^−/−^-pup ileum and **b** colon; **c** Different effects of postbiotics on TjP1 mRNA expression in C57-pup and TLR4^−/−^-pup ileum. Data are represented as the mean ± SEM (*n* = 12–14). One-way ANOVA and Tukey post hoc tests were used for statistical analysis. Differences were considered significant at *P < 0.05, **P < 0.01, ***P < 0.001 levels.

Figure 5 shows the effects of *B. infantis* postbiotics on Ki67 and TjP1 mRNA expression in wild-type (WT) C57BL/6-pups and TLR4^−/−^-pups in an intestinal range dependent manner (ileum vs. colon). In untreated TLR4^−/−^ mice, Ki67 mRNA expression in the ileum was lower (but did not reach statistical significance) when compared with WT mice (Fig. 5a), while no difference was detected in the colon (Fig. 5b). Postbiotics exposure significantly increased Ki67 mRNA expression in both ileum (Fig. 5a) and colon (Fig. 5b) in WT mice, but not in the TLR4^−/−^ mice. Similarly, TjP1 mRNA expression was significantly decreased in the ileum of untreated TLR4^−/−^ mice compared to WT mice (Fig. 5c). Postbiotic exposure increased TjP1 mRNA expression in both WT and TLR4^−/−^ mice, however its expression in WT mice remained significantly higher when compared to TLR4^−/−^ mice (Fig. 5c).

### TLR4 regulates the mRNA expression of cytoskeleton genes induced by postbiotics from B. infantis in mouse pups

**Fig. 6:**
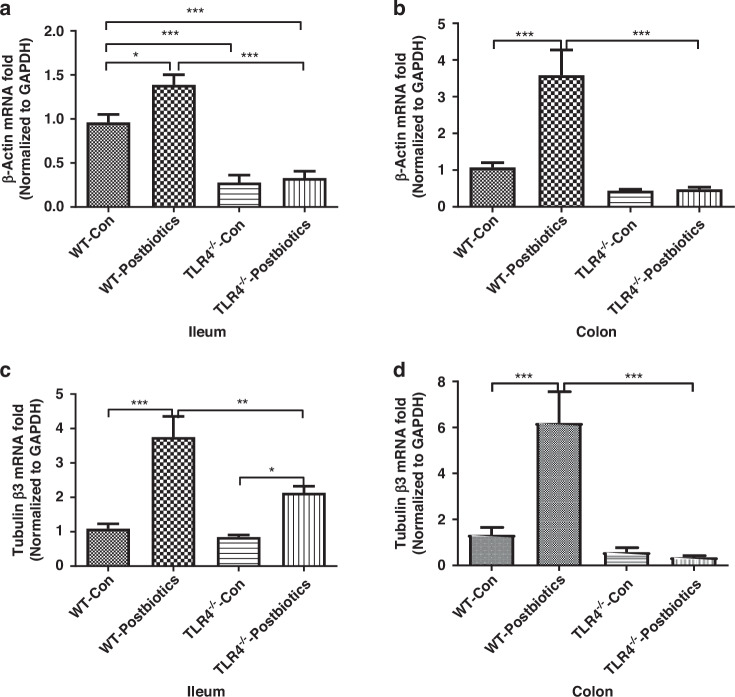
TLR4 regulates the mRNA induction of β-Actin and Tubulin β3-induced by postbiotics from *B. infantis* in mouse pups. **a** Different effects of postbiotics on β-Actin mRNA expression in C57-pup and TLR4^−/−^-pup ileum and **b** colon; **c** Different effects of postbiotics on Tubulin β3 mRNA expression in C57-pup and TLR4^−/−^-pup ileum and **d** colon. Data are represented as the mean ± SEM (*n* = 12–14). One-way ANOVA and Tukey post hoc tests were used for statistical analysis. Differences were considered significant at *P < 0.05, **P < 0.01, ***P < 0.001.

We then explored the TLR4-dependent effect, both at baseline and after *B. infantis* postbiotics exposure, on the gene expression of β-Actin and Tubulin β3, two key components of the cell cytoskeleton. In untreated animals, mRNA expression of *β-Actin* was higher in WT mice compared to TLR4^−/−^ mice in the ileum (Fig. 6a), while no difference was detected in the colon (Fig. 6b). Exposure to *B. infantis* postbiotics caused an upregulation of *β-Actin* both in the ileum (Fig. 6a) and colon (Fig. 6b), in WT mice but not in TLR4^−/−^ mice (Fig. 6a, b). In untreated mice, we detected no difference in *Tubulin β3* gene expression between WT and TLR4^−/−^ mice, both in the ileum (Fig. 6c) and the colon (Fig. 6d). In the ileum, treatment with *B. infantis* postbiotics increased *Tubulin β3* gene expression both in WT and TLR4^−/−^ mice, albeit it was significantly lower in TLR4^−/−^ mice compared to WT mice **(**Fig. 6c). In the colon, treatment with *B. infantis* postbiotics caused upregulation of *Tubulin β3* gene expression only in WT mice but not TLR4^−/−^ mice (Fig. 6d).

### TLR4 altered the effects of B. infantis postbiotics on Krt18 mRNA, Krt20 mRNA in wild-type and TLR4^−/−^ mouse pups

**Fig. 7:**
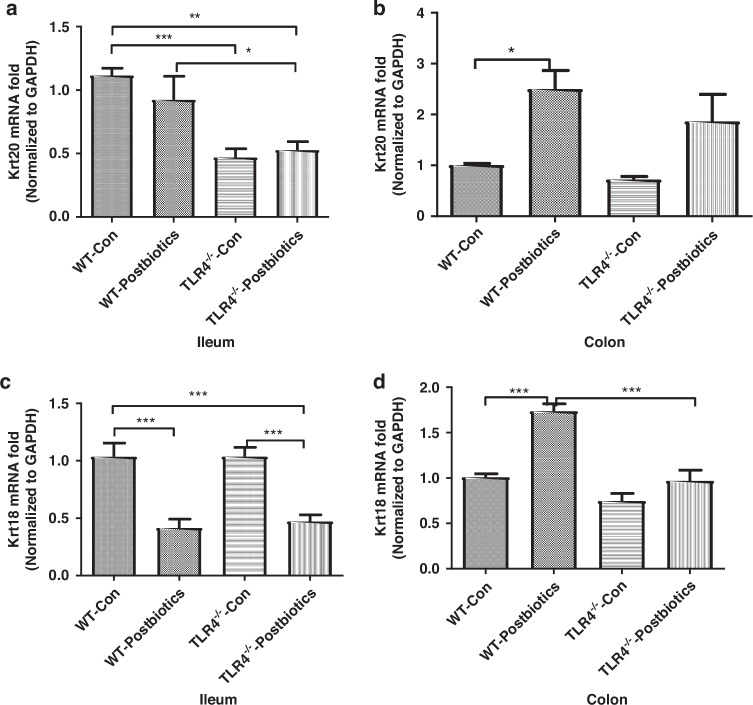
TLR4 regulates the effects of postbiotics from *B. infantis* on Krt20 mRNA in wild-type and TLR4^−/−^ mouse pups. **a** Differential effect of postbiotics on Krt20 mRNA expression in C57-pup and TLR4^−/−^-pup ileum; **b** Differential effect of postbiotics on Krt20 mRNA expression in C57-pup and TLR4^−/−^-pup colon. **c** Differential effect of postbiotics on Krt18 mRNA expression in C57-pup and TLR4^−/−^-pup ileum; **d** Differential effect of postbiotics on Krt18 mRNA expression in C57-pup and TLR4^−/−^-pup colon. Data are represented as the mean ± SEM (*n* = 12–14). One-way ANOVA and Tukey post hoc tests were used for statistical analysis. Differences were considered significant at *P < 0.05, **P < 0.01, ***P < 0.001.

Finally, we analyzed the gene expression of Krt18 and Krt20, key growth-associated proteins important for cell maturation. In the ileum, Krt20 gene expression was higher in untreated WT mice compared to TLR4^−/−^ mice (Fig. 7a), while no difference was detected in the colon (Fig. 7b). When exposed to *B. infantis* postbiotics, no changes were detected in the ileum of both WT and TLR mice (Fig. 7a), while an increase in *Krt20* gene expression was detected in the colon of WT but not TLR4^−/−^ mice (Fig. 7b). *Krt18* gene expression was similar in both untreated WT and TLR4^−/−^ mice (both ileum and colon), while treatment with postbiotics caused an upregulation of *Krt18* gene expression only in the colon of WT mice but not TLR4^−/−^ mice. Downregulation of *Krt18* gene expression was observed in the ileum of both WT and TLR^−/−^ mice with postbiotic administration.

## Discussion

The premature infant is at risk for adverse medical conditions, particularly inflammation, because of its inability to appropriately adapt to the extra-uterine environment. In the latter stages of gestation, maturation of the gastrointestinal tract is required for the term infant to navigate challenges in the outside world which includes a rapid colonization of the gastrointestinal tract by microorganisms, both beneficial as well as harmful. The lack of this normal fetal maturation due to preterm birth increases the risk of development of a necrotizing inflammation of the distal small intestine and colon, known as necrotizing enterocolitis (NEC).^1–4,21,22^ Almost ten percent of infants under 1500 grams develop this condition.^23,24^

Accordingly, research in this area has targeted prevention of this debilitating condition by assisting prematures to develop both immunologic and metabolic means of coping with the environment. An important contributor to this process is the TLR4 receptor which has been implicated in excessive inflammation in the first two weeks of extrauterine life, a period during which bacterial colonization occurs. As a result, excessive expression of this receptor on enterocytes due to colonizing bacteria under dysbiotic conditions can cause excessive inflammation of the gut leading to NEC.^24–26^ However, we and others have reported that certain bacteria, *Lactobacillus* and *Bifidobacteria*, particularly *B. infantis* and its metabolites, can also act as probiotics to stimulate an anti-inflammatory response to counter excessive inflammation, and hence to prevent NEC.^1,2,5^ These anti-inflammatory conditions associated with probiotics require the presence of the TLR4 receptor.^12,13^

The extent to which TLR4 is also involved in gastrointestinal development in the immediate postpartum period is the subject of this report. A previous study has shown that TLR4 appear on crypt cells, implying but not proving a role in development in gestation.^27^ We also know that the Drosophila TLR4-equivalent receptor has these additional functions during development.^15^ In the mature organism, the TLR4-equivalent functions as an innate immune receptor whereas during maturation it also assumes developmental functions.^15,27,28^ We provide in vitro evidence using a fetal human intestinal cell line, authenticated by organoids from two fetal intestines at ages 15 and 21 weeks with similar results (21 weeks is not shown). We also showed that in intestine of mouse pups, the TLR4 receptor is necessary to stimulate cell proliferation, cytoskeletal modification, and intestinal barrier development. The evidence to support this thesis is provided below.

H4 cells, a well-characterized human fetal small intestinal epithelial cell line^16^ and mouse model have been extensively used as a cellular tool for studies of the inflammatory response and the pathophysiology of NEC.^13,14^ After 4 days, with formula feeding and at low oxygen levels, wild-type mice can develop patchy intestinal edema, inflammation, and necrosis which closely resembles human NEC.^26^ In the present study, H4 cells and wild-type C57BL/6-pups on postnatal day 4 were used to investigate the role of postbiotics (postbiotic conditional media) from *B. infantis* on intestinal epithelium development. In contrast, TLR4 gene knock down H4 (H4-TLR4KD) cells and TLR4 gene knockout (TLR4^−/−^) neonatal mice were used to determine the role of TLR4 with postbiotic stimulation. The results both in vitro and in vivo suggest that TLR4 is required at least in part for postbiotic-induction of cell proliferation, cytoskeleton modification, and intestinal barrier development.

Ki67 is a nonhistone nuclear protein involved in cell proliferation. Ki67 protein is present during the active phases of the cell cycle. Its function is closely correlated with mitosis and is indispensable in cell proliferation.^17^ Here, postbiotics increased both Ki67 mRNA expression and protein expression of immature human enterocytes in vitro (Figs. 1 and 2) while postbiotics increased only Ki67 mRNA expression of the immature mouse intestine in vivo (Fig. 5) (protein data are not shown). TLR4 gene knockdown or knockout inhibited the postbiotic-induced *Ki67* gene and protein induction in vitro (Figs. 1a and 2) and Ki67 mRNA induction in vivo (Fig. 5a, b). Although, the Ki67 protein level is higher in small intestinal tissue in TLR4^−/−^ mice compared to WT mice, postbiotics did not affect Ki67 protein levels in both WT and TLR4 ^−/−^ mice. Our results indicated that postbiotic- induced intestinal epithelial cell proliferation was in part dependent on the TLR4 signaling pathway in both human and mouse intestine. Previous studies reported that human milk oligosaccharides efficiently protected against NEC by restoring the proliferative ability of enterocytes in the ileum which increased the ratio of Ki67 expression by reducing the expression of TLR4 on intestinal epithelial cells.^29^ Weber et al. also reported that the proliferation marker Ki67 expression decreased in TLR4-deficient compared to TLR4-sufficient mice^30^ suggesting a proliferative state dependent on TLR4 signaling whose mechanism was similar to ours in this study.

Telomerase reverse transcriptase (TERT, or hTERT in humans) is a catalytic subunit of the enzyme telomerase. Telomerase maintains structures called telomeres which are composed of repeat segments of DNA found at the ends of chromosomes to protect chromosomes from inappropriately sticking together or degrading.^31^ In most cells, telomeres become progressively shorter as the cell divides. Telomerase counteracts the shortening effect of telomeres by adding small repeat segments of DNA to the ends of chromosomes each time the cell divides.^31,32^ TERT expression is also representative of an active cellular proliferation situation.^31,32^ In this study, postbiotics significantly increased hTERT mRNA expression in immature H4 enterocytes in a TLR4-dependent manner suggesting that the high hTERT mRNA activity also contributed to postbiotic-induced H4 cell proliferation (Fig. 1b).

Cytoskeleton proteins provide mechanical support for cells and provide transport pathways through the cytoplasm to facilitate the rapid assembly and disassembly of signal transduction actin networks, enabling cells to divide and migrate. Postbiotics appeared to modify cytoskeleton proteins F-actin, β-actin, and Tubulin β3 in this study (Figs. 3 and 6). Actin is the major constituent of the cellular cytoskeleton. Actins are highly conserved proteins in monomeric form as G-actin (globular actin) or can form filaments (F-actin, filamentous actin) and mediates multiple cellular functions. β-actin, also known as cytoplasmic actin, are predominantly expressed in non-muscle cells, controlling cell motility, structure, and integrity. The rapid assembly and disassembly of actin networks enables cells to migrate and actin filaments can facilitate signal transduction.^33^ Tubulin is a major building block of microtubules whose intracellular cylindrical filamentous structure is present in almost all eukaryotic cells with essential roles in cell division, shape, motility, and intracellular transport. Tubulin-β3 is an important subunit isotype.^34,35^ Here, postbiotics upregulated Tubulin-β3 mRNA expression levels in immature intestinal tissues indicating that the early life colonizing bacteria *B. infantis* plays an important role in intestinal development. Moreover, we found tubulin (Fig. 6c, d) is developmentally regulated in the intestine and TLR4 negatively regulates tubulin expression in immature mouse intestine (Fig. 6c, d). Previous studies also reported differential requirements of tubulin genes in mammalian forebrain and neuronal development.^36,37^ Keratins are a family of structural proteins that form the intermediate filaments of the cytoskeleton in intestinal epithelial cells while embryonic keratin type Krt18 is predominant in the undifferentiated crypt compartment and adult keratin type Krt20 is predominantly detectable in the villus as a gastrointestinal differentiation marker.^38,39^ The different effects of postbiotics on embryonic and adult keratin genes further confirm *B. infantis*’ induction of intestine maturation and the developmental regulation role of TLR4 (Fig. 7).

The high incidence of bacterial translocation in newborns is thought to be caused partially by immaturity of the intestinal mucosal barrier function. Tight junction proteins (TJP) form a continuous barrier to fluids passing across the epithelium and endothelium which function in regulation of paracellular permeability, the maintenance of cell polarity, and the blocking the movement of transmembrane proteins between the apical and the basolateral cell surfaces.^40^ Zona occludens (ZO) are peripheral membrane adaptor proteins that link junctional transmembrane proteins.^18^ ZO-1, also known as TjP1, is a protein located on a cytoplasmic membrane surface of intercellular tight junctions and is required for tight junction formation and function which also participate in signal transduction mechanisms that regulate epithelial cell proliferation, gene expression, differentiation, and morphogenesis, possibly through facilitating nuclear import/export of transcriptional regulators.^18,19^ Our results indicated that *B. infantis* postbiotics improved the tight junction in immature intestine both with and without TLR4 (Figs. 4 and 5c).

Probiotic-conditional media components (postbiotics) provide an additional benefit to the supplementation of *B. infantis* in premature human infants in order to adapt to the extra-uterine environment and to prevent NEC. Short-chain fatty acids^9^ and Indole-3-lactic acid (ILA)^10^ are two postbiotic components which actively interact with the underlying intestinal mucosa to facilitate developmental adaptation. Although these undoubtedly are additional postbiotic molecules that help accomplish this developmental function, the observation provides additional reason to consider postbiotic supplementation in the preventive treatment of premature infants.

## Conclusion

In this study, we provide evidence that the TLR4 receptor is involved in part with intestinal development of premature human infants in a manner similar to that described in Drosophila during development. The TLR4 receptor is partially responsible for the impact of postbiotics from *B. infantis* in stimulating both genes and proteins in vitro and in vivo for development. This observation may provide evidence in support of the use of preterm breast milk and the probiotic strain *B. infantis* for the prevention of NEC.

The datasets generated and analyzed during the current study are available in the Figshare repository at 10.6084/m9.figshare.26213747

## Data Availability

The datasets generated and analyzed during the current study are available in the Figshare repository at 10.6084/m9.figshare.26213747.
